# Prolonged hemidiaphragmatic paresis following continuous interscalene brachial plexus block: A case report

**DOI:** 10.1097/MD.0000000000003891

**Published:** 2016-06-17

**Authors:** Helen Ki Shinn, Byung-Gun Kim, Jong Kwon Jung, Hee Uk Kwon, Chunwoo Yang, Jonghun Won

**Affiliations:** a Department of Anesthesiology and Pain Medicine, Inha University School of Medicine, Incheon; b Department of Anesthesiology and Pain Medicine, Konyang University School of Medicine, Daejeon, Republic of Korea.

**Keywords:** hemidiaphragmatic paresis, interscalene block, nerve injury, phrenic nerve

## Abstract

Interscalene brachial plexus block provides effective anesthesia and analgesia for shoulder surgery. One of the disadvantages of this technique is the risk of hemidiaphragmatic paresis, which can occur as a result of phrenic nerve block and can cause a decrease in the pulmonary function, limiting the use of the block in patients with reduced functional residual capacity or a preexisting pulmonary disease. However, it is generally transient and is resolved over the duration of the local anesthetic's action.

We present a case of a patient who experienced prolonged hemidiaphragmatic paresis following a continuous interscalene brachial plexus block for the postoperative pain management of shoulder surgery, and suggest a mechanism that may have led to this adverse effect.

Nerve injuries associated with peripheral nerve blocks may be caused by several mechanisms. Our findings suggest that perioperative nerve injuries can occur as a result of combined mechanical and chemical injuries.

## Introduction

Shoulder surgery usually results in moderate to severe postoperative pain that can impair early recovery and rehabilitation. Single-injection or continuous interscalene brachial plexus block (ISB) provides effective anesthesia and analgesia for shoulder surgery. However, one of the disadvantages of this technique is the risk of hemidiaphragmatic paresis (HDP) as a result of ipsilateral phrenic nerve blocks and the potential decrease in the pulmonary function,^[1,2]^ which may limit the use of the technique in patients with reduced functional residual capacity or a preexisting pulmonary disease. It may also be a serious concern for ambulatory patients.^[3]^ However, this type of paresis is generally transient and is resolved over the duration of the local anesthetic's action.

We report an unusual case of prolonged HDP following continuous ISB for shoulder surgery that was almost fully recovered after 15 months. This report discusses the etiology and mechanism of this rare perioperative nerve injury.

## Case report

**Figure 1:**
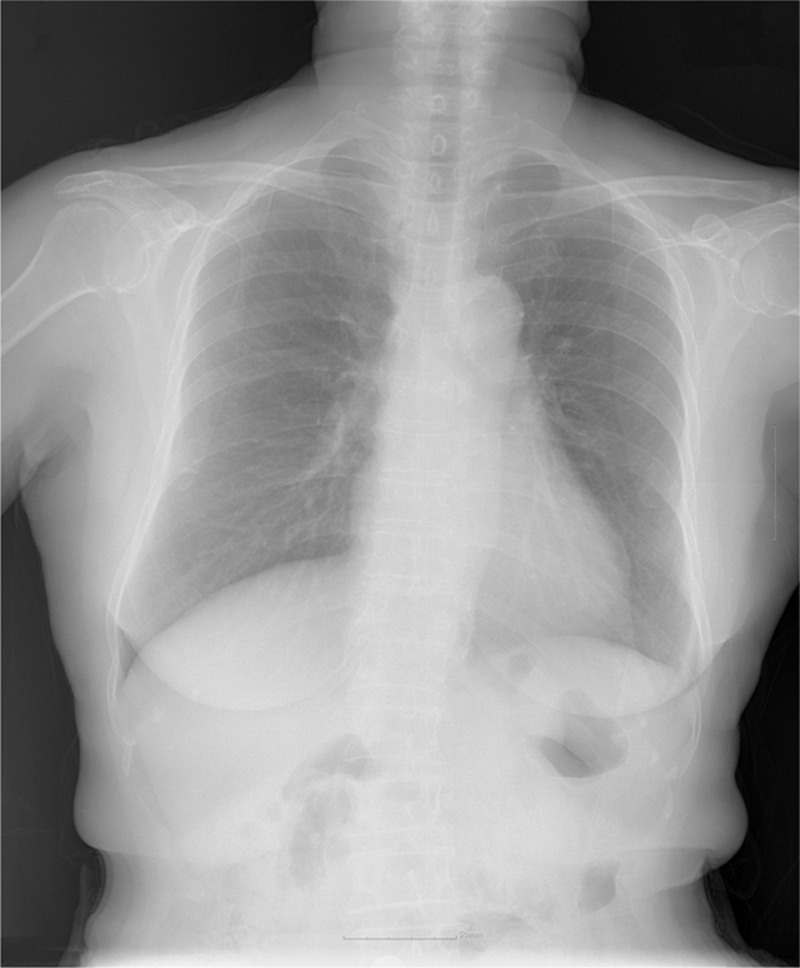
Preoperative chest X-ray.

A 71-year-old woman of American Society of Anesthesiologists physical status II was scheduled for arthroscopic rotator cuff repair of the right shoulder under ISB. Her only remarkable medical history was hypertension treated with carvedilol 12.5 mg, PO daily. She had no history of cervical spine or lung disease and no preexisting neurologic deficits. The preoperative laboratory evaluations – including her chest radiograph (Fig. 1) – were unremarkable.

After applying standard monitoring, fentanyl 50 μg was administered to produce a state of conscious sedation. No superficial cervical plexus block was performed for shoulder analgesia. A right continuous ISB was performed using a similar technique to the one described before.^[4]^ Following skin disinfection, a skin wheal with 3 mL of 2% lidocaine was raised to the needle entry point. A nerve stimulator (Stimuplex-DIG, B/Braun, Melsungen, Germany) connected to a 50 mm insulated Contiplex A needle and cannula (Contiplex A; B/Braun) were used with the following initial settings: frequency = 1 Hz, stimulus duration = 0.2 ms, and current = 1.5 mA. On the 1st pass, we noticed hiccups from diaphragm contractions, and the needle was redirected laterally and posteriorly. On the 2nd pass, a contraction of the biceps muscle was observed under a current output of 0.5 mA. During the introduction of the cannula, the patient reported a brief paresthesia of the shoulder area that was quickly resolved. A few seconds later, as the paresthesia had rapidly disappeared, a 20-gauge end-hole catheter was inserted caudally by 8 cm through the cannula into the plexus sheath without dysesthesia or pain. Subsequently, 30 mL of 0.5% ropivacaine were slowly injected through the catheter after careful intermittent aspirations. Thirty minutes after the initial bolus, the patient exhibited a sensory and motor blockade without any subjective increase in breathing efforts. The catheter was then tunneled subcutaneously by 3 to 4 cm through an 18-gauge IV needle and was fixed to the skin with a tight suture. The patient was placed in the beach chair for the surgery. Her head was firmly fixed in the neutral position with a strap. A propofol infusion was administered intraoperatively for sedation. The 195 minutes of surgery was uneventful. In the recovery room, a 0.2% ropivacaine infusion was administered through the catheter at a rate of 6 mL/hour with a 3-mL bolus and a lockout of 20 minutes. The patient showed excellent analgesia and required no supplemental analgesics.

By the following afternoon (30 hours after the initial block), the catheter was inadvertently dislodged. The patient's shoulder became progressively painful, and she requested that the interscalene catheter be reinserted. A 2nd continuous ISB was performed using the same technique as before, except that 20 mL of 0.2% ropivacaine were used for the analgesia this time. No phrenic nerve stimulation, pain, dysesthesia, or paresthesia occurred during the procedure. The patient underwent a routine chest radiograph to exclude the presence of a pneumothorax. The chest radiograph revealed an elevated ipsilateral hemidiaphragm with no other findings. At that time, there were no breathing problems reported. The local anesthetic infusion was discontinued on postoperative day 4. The catheter was removed without difficulty. There were no bruises, swelling, or infection at the insertion site. No neurologic deficits were noted in the patient's right upper extremity, and she was discharged on the 5th postoperative day.

**Figure 2:**
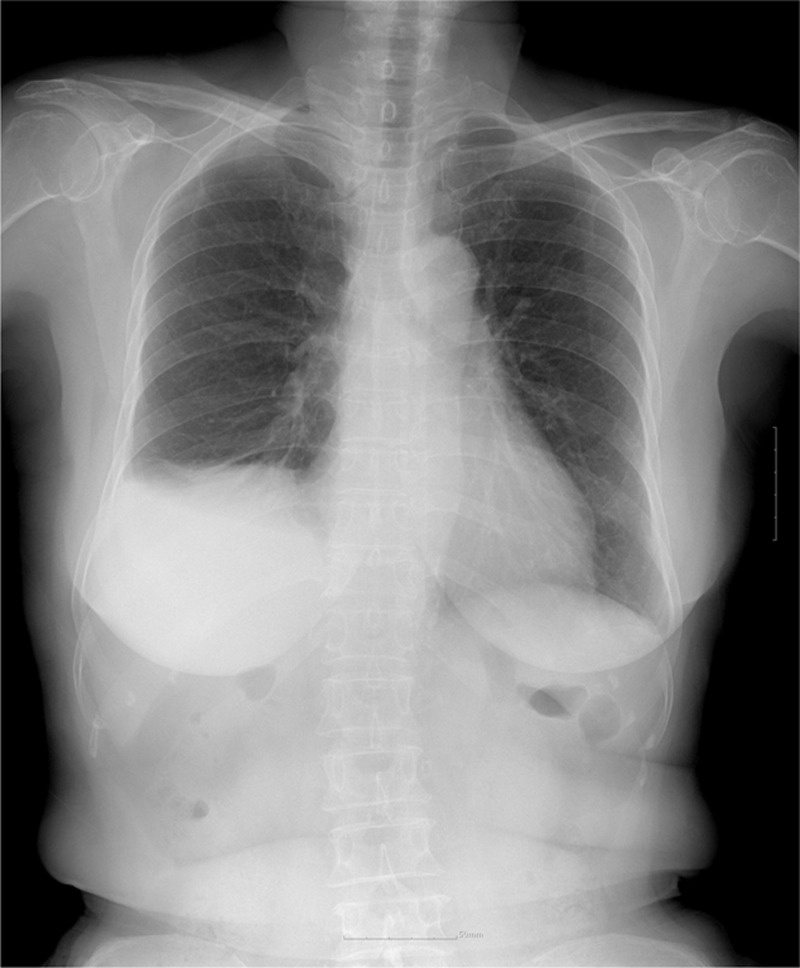
Chest X-ray at 3 months showing an elevation of the right hemidiaphragm associated with basal atelectasis of the right lower lobe and pleural effusion.

At the 2nd follow-up, approximately 3 months later, the patient complained of mild dyspnea, particularly upon bending of the spine. She was referred to a pulmonologist for further evaluation. The repeat chest radiograph reaffirmed an elevated right hemidiaphragm with a basal atelectasis and pleural effusion (Fig. 2). The pulmonary function tests showed a restrictive lung disease: the forced vital capacity, forced expiratory volume in 1 second, and peak expiratory flow were respectively reduced to 62%, 60%, and 69%. The bronchoscopy showed no abnormal findings. The thoracic computed tomography confirmed an elevation of the right diaphragm and collapses of the right lower lobe without any bronchial obstruction lesion. The patient was examined by the anesthesiologist. Unfortunately, no formal neurology consultation or neurophysiological studies were performed at that time.

**Figure 3:**
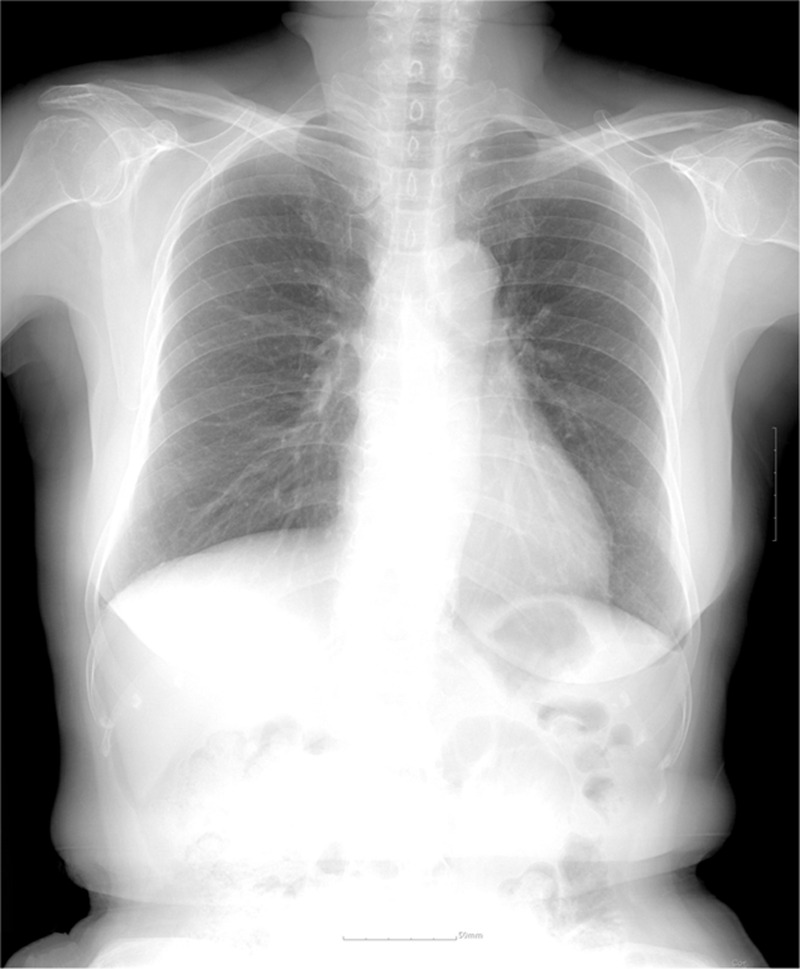
Chest X-ray at 15 months postoperatively showing fully expanded lungs.

Approximately 7 months later, the patient exhibited no further respiratory symptoms. She did not experience any interference in her normal daily activities from the HDP. The follow-up chest radiograph showed a mild improvement of the right lower lobe atelectasis. In the final follow-up, almost 15 months postoperatively, the chest radiograph had completely returned to the baseline (Fig. 3).

## Discussion

HDP from ipsilateral phrenic nerve block is a well-known side effect of ISB. When using the single-injection technique, ISB is associated with a 100% incidence of HDP, which can result in a 25% to 32% reduction in the spirometric measures of the pulmonary function.^[2]^ This side effect is not considered to be worrying unless the patient experiences a severe respiratory compromise or coincident contralateral phrenic nerve paresis. Patients undergoing continuous ISB are also at risk of HDP.^[5]^ Although HDP is usually transient, rare cases of prolonged or persistent HDP after single-injection or continuous ISB have been reported.^[6–8]^ The incidence of persistent HDP following ISB for shoulder surgery is of 1 per 2069 (0.048%).^[9]^

Transient HDP following ISB is presumably due to the ventral spread of the local anesthetic to the phrenic nerve, or the cephalad spread to the C3–5 roots of the cervical plexus. Therefore, it is usually resolved with the resolution of the anesthetic block. By contrast, the exact mechanisms of prolonged or persistent HDP remain unknown. Neurologic injuries can be caused by patient-, block-, or surgery-related factors, or a combination of those. The potential factors include diabetes mellitus, pre-existing neurologic disorders, being of the male gender, direct mechanical trauma, local anesthetic neurotoxicity, an ischemic injury, nerve compression from hematoma, and patient positioning or manipulation during the surgery. They may occur either alone or in combination. A recent study reported that symptomatic cervical spine disease was a risk factor of persistent HDP following ISB.^[9]^

In our case, the mechanism of the nerve injury was uncertain and probably multifactorial. The patient had no history of cervical spine disease or chiropractic manipulation. Although traction injuries on the operated extremity may occur during surgical procedures, they are primarily associated with the lateral decubitus position.^[10]^ In addition, the head was fixed firmly in the neutral position throughout the surgical procedure. Therefore, it is unlikely that the paresis was caused by the surgery or patient positioning in this case.

Given the course of the events, it is more likely that the paresis was caused by the continuous ISB. Our patient reported paresthesia in the operated shoulder during the cannula insertion. Although mechanical contact with the brachial plexus generally results in shoulder paresthesia, it can also be caused by the stimulation of the phrenic nerve. Therefore, the insertion of the cannula may have been one of the factors that contributed to the nerve injury in this case. However, it is unknown whether the elicitation of paresthesia increases the risk of nerve injuries.

The presence of an indwelling catheter might be a factor of nerve injuries. Indeed, it is possible that it causes fibrosis around the nervous tissue.^[11]^ A recent study suggested that inflammation played a significant role in the mechanism of persistent HDP.^[8]^ The study reported intraoperative findings of adhesions, fascial thickening, and vascular changes secondary to inflammation during phrenic nerve surgery. However, although inflammation might play a crucial role in the mechanism of nerve injuries, it is unclear whether this results from the catheter or from other factors.

With regard to the phrenic nerve injury in the present case, the potential chemical toxicity of the local anesthetics must be considered. Although local anesthetics are generally safe when administered correctly and in the recommended concentrations, they can produce nerve injuries, particularly in the event of inappropriately high concentrations, intraneural injections, or a prolonged exposure duration. In the present case, ropivacaine 0.2% was infused continuously for several days. Therefore, the neurotoxicity of the local anesthetics might also have contributed to the paresis, although ropivacaine seems to present the least potential for neurotoxicity of all available local anesthetics.

In this case, the brachial plexus was located through electrical stimulation. We did not use ultrasound, as we did not have the necessary equipment at the time. The advantages of ultrasound-guided nerve blocks include the real-time visualization of the needle placement and avoidance of needle trauma. Although this might theoretically reduce the incidence of neurologic complications, there is no evidence that ultrasound guidance reduces the incidence of neurologic complications as compared with other techniques. In addition, permanent phrenic nerve injuries can occur despite the use of ultrasound.^[8]^ This suggests that direct mechanical trauma is not the main contributor to nerve injury.

The degree to which a nerve is damaged has implications for its function and potential recovery. Considering the clinical pathway in this case, the injury was more likely to represent neurapraxia or a mild degree of axonotmesis. Unfortunately, neurophysiological testing was not performed in this case. Neurophysiological testing is helpful to determine the basis of any clinical deficit, localize the site of the lesion, and define its severity and prognosis. Although neurophysiological testing does not indicate the etiology of the nerve injury, it may help to distinguish between various possibilities. The timing of the neurophysiologic studies is important in order to obtain an accurate diagnosis. Although the process of nerve degeneration may take 14 days or more, earlier testing is indicated to rule out preexisting neuropathies.

## Conclusion

This case report suggests that prolonged HDP can occur as a result of combined mechanical and chemical injuries following continuous ISB. A complete remission of the symptoms occurred spontaneously after 15 months. More research is needed on the exact mechanisms of nerve injuries following nerve blockades, and the methods to prevent them.

## References

[1] Urmey WF Talts KH Sharrock NE One hundred percent incidence of hemidiaphragmatic paresis associated with interscalene brachial plexus anesthesia as diagnosed by ultrasonography. Anesth Analg 1991;72:498–503. 2006740 10.1213/00000539-199104000-00014

[2] Urmey WF McDonald M Hemidiaphragmatic paresis during interscalene brachial plexus block: effects on pulmonary function and chest wall mechanics. Anesth Analg 1992;74:352–7. 1539813 10.1213/00000539-199203000-00006

[3] Ediale KR Myung CR Neuman GG Prolonged hemidiaphragmatic paralysis following interscalene brachial plexus block. J Clin Anesth 2004;16:573–5. 15610837 10.1016/j.jclinane.2004.03.005

[4] Yang CW Jung SM Kwon HU A clinical comparison of continuous interscalene brachial plexus block with different basal infusion rates of 0.2% ropivacaine for shoulder surgery. Korean J Anesthesiol 2010;59:27–33. 20651995 10.4097/kjae.2010.59.1.27 PMC2908223

[5] Yang CW Jung SM Cho CK Pleural effusion and atelectasis during continuous interscalene brachial plexus block -A case report. Korean J Anesthesiol 2010;58:95–8. 20498819 10.4097/kjae.2010.58.1.95 PMC2872898

[6] Bashein G Robertson HT Kennedy WF Jr Persistent phrenic nerve paresis following interscalene brachial plexus block. Anesthesiology 1985;63:102–4. 4014757 10.1097/00000542-198507000-00017

[7] Robaux S Bouaziz H Boisseau N Persistent phrenic nerve paralysis following interscalene brachial plexus block. Anesthesiology 2001;95:1519–21. 11748414 10.1097/00000542-200112000-00035

[8] Kaufman MR Elkwood AI Rose MI Surgical treatment of permanent diaphragm paralysis after interscalene nerve block for shoulder surgery. Anesthesiology 2013;119:484–7. 23838708 10.1097/ALN.0b013e31829c2f22

[9] Pakala SR Beckman JD Lyman S Cervical spine disease is a risk factor for persistent phrenic nerve paresis following interscalene nerve block. Reg Anesth Pain Med 2013;38:239–42. 23518866 10.1097/AAP.0b013e318289e922

[10] Rains DD Rooke GA Wahl CJ Pathomechanisms and complications related to patient positioning and anesthesia during shoulder arthroscopy. Arthroscopy 2011;27:532–41. 21186092 10.1016/j.arthro.2010.09.008

[11] Duclas R Jr Robards CB Ladie BL Tip adhesions complicate infraclavicular catheter removal. Can J Anaesth 2011;58:482–3. 21359616 10.1007/s12630-011-9473-y

